# Mental health and quality of life in patients with chronic liver disease: a single-center structural equation model

**DOI:** 10.1186/s12876-024-03268-x

**Published:** 2024-06-05

**Authors:** Sara Rizvi Jafree, Ammara Naveed, Humna Ahsan, Syeda Khadija Burhan, Masha Asad Khan, Amna Khawar, Florian Fischer

**Affiliations:** 1 Department of Sociology, Forman Christian College University, Lahore, Pakistan; 2 Pakistan Kidney Liver Institute and Research Centre, Lahore, Pakistan; 3 Department of Economics, Forman Christian College University, Lahore, Pakistan; 4 Department of Education, Forman Christian College University, Lahore, Pakistan; 5 Academic Dean of Humanities and Social Sciences, Kinnaird College for Women, Lahore, Pakistan; 6 Department of Psychology, Lahore College of Women University, Lahore, Pakistan; 7 Institute of Public Health, Charité – Universitätsmedizin Berlin, Berlin, Germany

**Keywords:** Chronic liver disease, Pakistan, Mental health, Quality of life, Coping

## Abstract

**Background:**

Chronic liver disease (CLD) is one of the leading disease burdens in Pakistan. Until now, there has only been limited focus in the country on providing health services through tertiary services in urban cities, whereas there is almost no research in Pakistan on the mental health and quality of life of CLD patients. This study aimed to understand which predictors influence the mental health and quality of life of CLD patients in order to advise better policy protection.

**Methods:**

Data was collected from CLD patients at the Pakistan Kidney and Liver Institute and Research Centre, Lahore, Pakistan. A total of 850 respondents were part of the final sample. The age of respondents ranged from 18 to 79 years and included the following diagnosis: (i) Chronic Viral Hepatitis (*n* = 271), (ii) Cirrhosis (*n* = 259), (iii) Hepatocellular Carcinoma (*n* = 193), and (iv) Non-viral Liver Disease (*n* = 127).

**Results:**

Mean results reveal that females as well as illiterate patients need more support for mental health and communication with their physician; whereas men need more support to develop coping strategies. Structural equation modelling results reveal that the severity of symptoms (β = 0.24, *p* < 0.001), coping strategies (β=-0.51, *p* < 0.001), and doctor communication (β=-0.35, *p* < 0.001) predict mental health. Quality of life is associated with the severity of symptoms (β=-0.36, *p* < 0.001), coping strategies (β = 0.26, *p* < 0.05), and doctor communication (β = 0.09, *p* < 0.05).

**Conclusions:**

A ‘bio-psycho-social-spiritual’ model is recommended for Pakistan’s CLD patients which includes the integration of social officers to provide support in four key areas to secure mental health and quality of life of patients.

## Background

Chronic liver disease (CLD) has become one of the most significant concerns of public health worldwide and is known to be one of the leading causes of death in the developing world [1]. Exact statistics for mortality and disability-adjusted life years are not available for regions like Asia and Africa due to lack of coordinated efforts for data collection [2]. Most of the studies conducted in Pakistan are hospital-based studies with small sample sizes and there is lack of community-based data or confirmed national data about the actual prevalence of CLD [3]. Despite the confirmation about prevalence, CLD has been classified as the fifth most common cause of morbidity and mortality in Pakistan and the country has been labelled by some as a “cirrhotic state” [4]. Yet, there is very little policy attention and investment for prevention or disease management of CLD to date in Pakistan [5].

Globally, there is great concern about the lack of support for mental health in patients suffering from CLD [6], and their quality of life [7]. In Pakistan specifically, there is even greater concern as mental health is not a priority area for the health sector [8], and the ‘bio-psycho-social-spiritual model’ [9] which is important to secure the quality of life in patients is less understood or integrated in the health system [10]. The bio-psycho-social-spiritual model was proposed by Engel (1977) as an inclusive model of healthcare which supports patients of chronic disease for not just biomedical care, but with care for the social, psychological, and behavioral dimensions of illness [11–13]. With gaps in statistics and limited efforts in Pakistan focused on delivering services to diagnosed patients of CLD in urban zones [14], there is even less empirical evidence about the mental health and quality of life of CLD patients. The aim of this study is to identify which predictors influence the mental health and quality of life of CLD patients in order to advise better policy protection for patients as they receive health services and support from family and providers. Data collected in this study has been analysed using structural equation modelling, which is less used in health research [15], even though it has benefits over the multivariate statistical techniques [16].

### Literature review

Quality of life in patients of CLD is affected primarily by the severity of symptoms, long period of treatment, and overall burden of care management [17, 18]. Common physical symptoms of patients with CLD include fatigue, inability to function at work, loss of appetite, and abdominal pain and swelling [19]. Personality changes have also been evidenced in CLD patients, such as loss of self-esteem, preference to remain isolated, and unwillingness to continue seeking health services [20, 21]. Patients of CLD are also known to suffer from common mental health challenges including anxiety, depression, constant worry, and even suicide ideation [22]. As severity of symptoms progresses in patients of CLD, their mental health and quality of life are known to decline [23]. Conversely, the occurrence of depressive indicators has an adverse impact on the progression of illness, reduced compliance with treatment, and quality of life [24, 25]. Local research also confirms that high levels of anxiety and depression are experienced by patients suffering from various liver diseases in Pakistan [26].

Effective doctor communication plays a salient role in CLD patients and their mental health and quality of life [27]. Comfort with doctor relations and good communication with physicians is known to facilitate increased awareness and understanding about the disease, timely check-up and testing, and compliance for health recovery [28]. Patients who do not have good communication with their physicians are known to suffer from additional stress and anxiety [29]. Local studies confirm that compliance of patients is strongly linked to empathetic communication and information sharing of doctors [30]. Previous research stresses that physicians need support for training in communication skills to improve quality services for patients and that their training needs are compromised due to low investment and prioritization by the health administration [31].

**Fig. 1:**
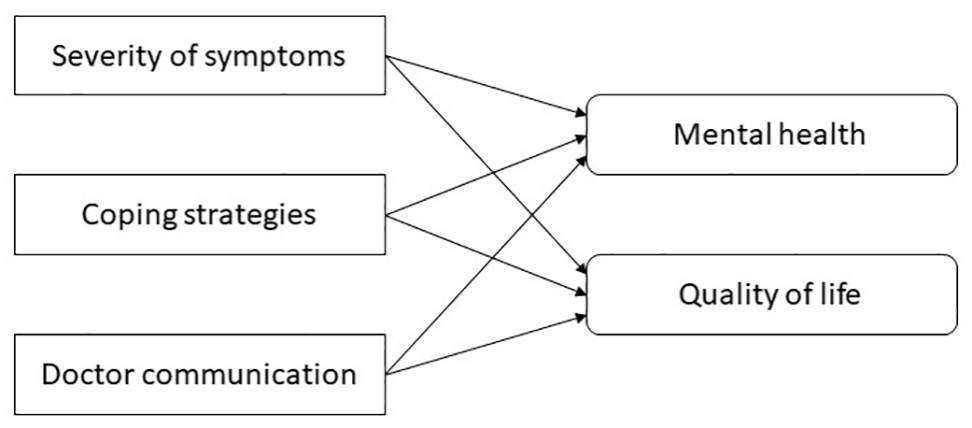
Conceptual model for the study

CLD management and recovery has a significant relationship with the patient’s overall quality of life [17, 32]. Along with the physical deterioration, and mental health impact, patients can suffer from socio-economic problems such as financial issues, lack of support from family and friends, and inability to continue with work [33, 34]. Some studies also highlight that low quality of life is associated with the stage of disease or number of symptoms, the level of liver function, and the extent of social support in patients [35]. During and in the aftermath of the COVID-19 pandemic, CLD patients have known to suffer a greater decline in quality of life due to a lack of clarity about how to manage their disease during and after the lockdown and how to deal with infection and vaccination protocols in a world with Coronavirus and its many variants [36]. The theoretical model for this study which is based on the previously described literature is summarized in Fig. 1.

### Aim and research questions

Much has been written about the causes of CLD, early screening, and preventive measures [37]. However, there is less research about the management and support of patients with CLD [38]. CLD is a progressive illness with high incidence of comorbidity, leading to decline in mental health and quality of life, necessitating holistic patient support. This study aims to bridge this gap and collect perception-based data from patients of CLD to identify which factors determine mental health and quality of life. Based on this study’s finding we would be able to advise more holistic care for patients of CLD in Pakistan.

The research questions for this study are:

(1) Which sociodemographic characteristics of patients with CLD (including age, gender, and literacy), are associated with severity of symptoms, coping strategies, doctor communication, mental health, and quality of life?

(2) What is the relationship between independent study constructs (severity of symptoms, coping strategies, doctor communication) with the dependent variables of mental health and quality of life?

### Study hypotheses

The following research hypotheses are tested in this study:

#### H1

Mental health has a positive relationship with severity of symptoms (i.e., the higher the disease symptoms, the greater the mental health challenges faced by patients of liver disease) and a negative relationship with coping strategies and doctor communication (i.e., the higher the coping strategies and the better the doctor communication, the lesser the mental health challenges faced by patients).

#### H2

Quality of life has a negative relationship with severity of symptoms (i.e., the greater the disease symptoms, the lesser the quality of life for patients of liver disease) and a positive relationship with coping strategies and doctor communication (i.e., the higher the coping strategies and the better the doctor communication, the better the quality of life of patients).

## Methods

### Research design

The study collected quantitative data, based on patients’ perceptions, through a closed-ended survey from the Pakistan Kidney and Liver Institute and Research Centre (PKLI&RC), Lahore, Pakistan. PKLI&RC is a single public centre dedicated for patients with liver diseases. Since its inception in December 2017, it has served 3.5 million patients, and almost 80% of them have received free or subsidized treatment [39].

### Sample

The selection criterion for this study was patients of CLD presenting to the outpatient Gastrointestinal Department of PKLI&RC for health services. Initial screening was done by the PKLI&RC doctors to exclude from the sample patients who were mentally infirm, delirious, or diagnosed with hepatic encephalopathy. Sampled patients fell under the following disease classifications: (i) Chronic Viral Hepatitis (*n* = 271), (ii) Cirrhosis (*n* = 259), (iii) Hepatocellular Carcinoma (*n* = 193), and (iv) Non-viral Liver Disease (*n* = 127). Data was not collected further on disease severity for cirrhotic patients, and parameters like CHILD, MELD and decompensation were not available to the research team.

### Data collection

Data was collected between May 2022 and August 2022 by experienced field researchers who were trained over a period of two weeks by the authors of this study. The survey was translated to Urdu, the local language through the forward and backward method by authors who are bilingual in English and Urdu [40]. All data was collected in a private space provided by PKLI&RC with data collectors assisting illiterate and semi-literate respondents for survey completion. The data has been analysed using SPSS and AMOS, considering a *p*-value less than 0.05 as significant.

### Instruments

#### Severity of symptoms

For this study, severity of symptoms is measured based on scores for three areas according to the Chronic Liver Disease Questionnaire [41]: Fatigue, abdominal symptoms, and systemic symptoms. A seven-point Likert scale was used to measure responses ranging from ‘All the time’ to ‘None of the time’.

Fatigue has been measured for this study using the following three items: ‘How much of the time have you been tired or fatigued during the last two weeks?’, ‘How much of the time in the last two weeks have you been bothered by having decreased strength?’, and ‘How often during the last two weeks have you felt a decreased level of energy?’.

Abdominal symptoms have been measured using the following three items: ‘How much of the time during the last two weeks have you been troubled by a feeling of abdominal bloating?’, ‘How much of the time during the last two weeks have you experienced abdominal pain?’ and ‘How much of the time during the last two weeks have you been troubled by a feeling of abdominal discomfort?’

Systemic symptoms have been measured using the following three items: ‘How much of the time during the last two weeks has shortness of breath been a problem for you in your daily activities?’, ‘How much of the time during the last two weeks have you been unable to fall asleep at night?’, and ‘How much of the time have you been troubled by itching during the last two weeks?’

#### Coping strategies

We used the COPE Inventory to measure coping [42]. Four items were used to measure religious coping: ‘I seek God’s help’, ‘I put my trust in God’, ‘I try to find comfort in my religion’, and ‘I pray more than usual’. Three items were used to measure active coping, with language modified to suit the study: ‘I take action to try to get the help I need’, ‘I concentrate my efforts on doing something about my disease’, and ‘I take measures to gain health/recovery’. Three items were used to measure planning strategies and language was modified to suit the study: ‘I try to come up with a strategy about what to do next to gain recovery/ health stability’, ‘I have a plan of action to manage my illness’, and ‘I think about how I might best handle my health problems’. A four-point Likert scale was used to measure responses ranging from ‘I usually do this a lot’ to ‘I usually don’t do this at all’.

#### Doctor communication

We used the QUOTE-liver scale to measure doctor communication quality [43]. The following three areas were measured: Knowledge, interaction and care. Knowledge was measured using the following three items: ‘Doctor is knowledgeable’, ‘Answers all questions’, and ‘Gives enough information about your disease/treatment’. Interaction was measured using the following three items: ‘Takes you seriously’, ‘Makes you feel safe’, and ‘Takes enough time for you’. Care was measured using the following three items: ‘Takes time to discuss emotional issues’, ‘Refers you well when you present with complaints that are not liver disease related’, and ‘Listens to you’. A four-point Likert scale was used to measure responses ranging from ‘Yes’, ‘Not really’, ‘Mostly Yes’ to ‘No’.

#### Mental health

We used the Instrument for Common Mental Disorders to measure mental health [44]. Four sub-domains were included: (i) Illness worry, (ii) anxiety, (iii) general fear and hopelessness, and (iv) depression. Illness worry was measured using the following three items: ‘Worry that there is something seriously wrong with your body’, ‘Worry about health all the time’, and ‘Thoughts that the doctor may be wrong in telling you not to worry’. Anxiety was measured using the following three items: ‘Feeling suddenly scared for no reason’, ‘Nervousness or shakiness inside’, and ‘Spells of terror and panic’. General fear and hopelessness was measured using the following three items: ‘Feeling fearful’, ‘Feeling hopeless about the future’, and ‘Feeling everything is an effort’. Depression was measured using the following three items: ‘Feelings of worthlessness’, ‘Thoughts of ending life’ and ‘feelings of being trapped or caught’. A five-point Likert scale was used to measure responses ranging from ‘Not at all’ to ‘Extremely’.

#### Quality of life

We used the brief WHOQOL survey to measure quality of life in CLD patients [45]. General quality of life was measured using three items: ‘How satisfied are you with the quality of your life?’, ‘In general, how satisfied are you with your life?’, and ‘How satisfied are you with your health?’. Level of independence was measured using the following three items: ‘How satisfied are you with your ability to perform your daily living activities?’, ‘How satisfied are you with your capacity for work?’, and ‘How satisfied are you with your ability to move around?’. A five-point Likert scale was used to measure responses ranging from ‘Very dissatisfied’ to ‘Very satisfied’.

### Reliability analysis

The Cronbach’s alpha (α) results for study variables have been reported in Table 1. All study variables have satisfactory Cronbach’s alpha values above 0.700 [46].

**Table 1:** Psychometric properties of study variables

Variable	α	Number of items
Severity of symptoms		
Fatigue Abdominal symptoms Systemic symptoms	0.840 0.795 0.813	3 3 3
Coping strategies		
Religious Active Planning	0.915 0.909 0.800	4 3 3
Doctor communication		
Knowledge Interaction Care	0.924 0.707 0.802	3 3 3
Mental health		
Illness worry Anxiety General fear and hopelessness Depression	0.926 0.839 0.814 0.885	4 3 3 3
Quality of life		
General quality of life Level of independence	0.711 0.830	3 3

### Ethics

This study received ethics clearance from the Ethics Review Committees of Forman Christian College University, and The Pakistan Kidney and Liver Institute and Research Centre (PKLI&RC), Lahore, Pakistan. Informed consent was taken from all respondents who participated in this study willingly.

## Results

### Sociodemographic characteristics

Table 2 summarizes the sociodemographic characteristics of participants. The majority of the 850 respondents of this study are above the age of 40 years (73.2%) and married (83.4%). The sample is split almost evenly between men and women and the majority have a monthly household income of USD 22.20–221.00 (66.7%), suggesting that most of the sample is from the lower middle class of Pakistan. Very few of the respondents have a graduate or postgraduate degree (14.7%) and few are currently employed (33.6%).

**Table 2:** Descriptive statistics of sociodemographic characteristics (*n* = 850)

Variable	*n* (%)
Age	
18–39 40–79	228 (26.8%) 622 (73.2%)
Gender	
Female Male	422 (49.6%) 428 (50.4%)
Marital status	
Currently married Unmarried	709 (83.4%) 141 (16.6%)
Literacy	
Illiterate Primary/secondary schooling Graduate/post-graduate	315 (37.1%) 410 (48.2%) 125 (14.7%)
Employment status	
Employed Unemployed	445 (33.6%) 394 (46.4%)
Monthly income	
PKR 5,000–49,999 (USD 22.20–221.00) PKR 50,000 and above (USD 222.00 and above)	567 (66.7%) 228 (33.4%)

### Prevalence of common mental health disorders

**Fig. 2:**
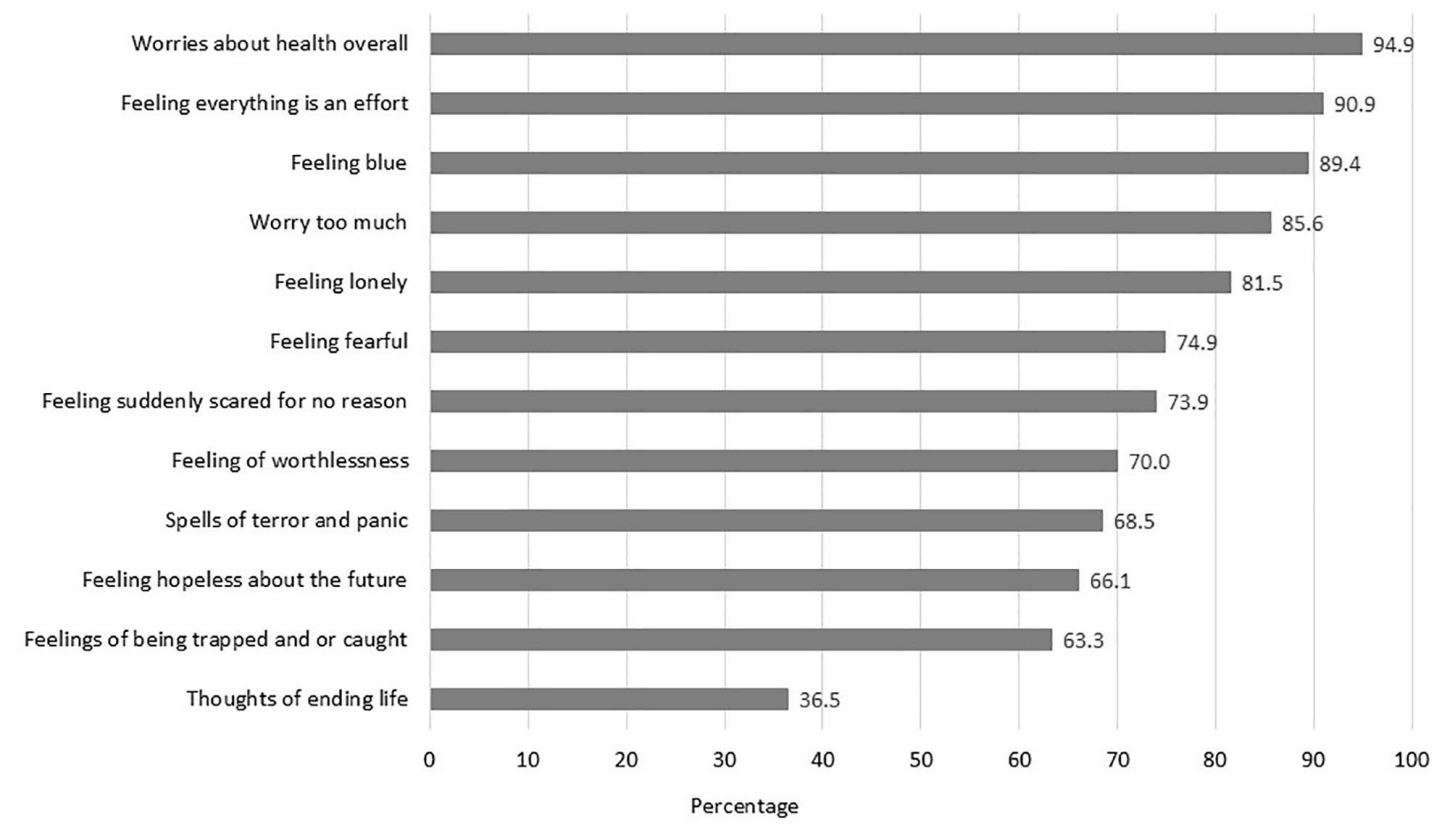
CLD patients’ experience of common mental health disorders

Respondents reported experiences in facing common mental health disorders (Fig. 2). The majority of CLD patients suffered from high incidence of worry about health overall (94.9%). Almost all patients felt that everything they do is an effort (90.9%), and felt blue or sad (89.4%). The majority also experienced loneliness (81.5%), fear (74.9%), and worthlessness (70.0%). More than half of respondents were hopeless about the future (66.1%) and felt trapped (63.3%). Of most concern is that almost half of CLD patients had thoughts of ending their life (36.5%).

### Mean comparisons

Mean results for age and study variables are presented in Table 3a. None of the results are significant.

**Table 3:** Independent sample t-tests between (a) age, (b) gender, and (c) literacy and study variables

**a) Age**				
***Variable***	***Age (in years)***	***Mean (SD)***	***t***	***p*** ***-value***
Severity of symptoms	18–39 40 and above	7.96 (3.60) 7.71 (3.81)	0.852	0.395
Coping strategies	18–39 40 and above	4.17 (1.46) 4.04 (1.36)	1.168	0.243
Doctor communication	18–39 40 and above	12.85 (2.62) 13.21 (2.62)	-1.728	0.084
Mental health	18–39 40 and above	9.63 (4.05) 9.53 (4.32)	0.305	0.760
Quality of life	18–39 40 and above	5.87 (1.69) 5.76 (1.95)	1.626	0.104
b) ***Gender***				
***Variable***	***Gender***	***Mean (SD)***	***t***	***p-value***
Severity of symptoms	Men Women	7.91 (3.81) 7.65 (3.69)	0.997	0.319
Coping strategies	Men Women	3.91 (1.31) 4.24 (1.45)	3.531	< 0.001
Doctor communication	Men Women	13.36 (2.37) 12.86 (2.83)	2.836	0.005
Mental health	Men Women	9.14 (4.42) 9.98 (4.03)	-2.878	0.004
Quality of life	Men Women	5.75 (1.97) 5.83 (1.78)	-1.418	0.157
c) ***Literacy***				
***Variable***	***Literacy***	***Mean (SD)***	***t***	***p-value***
Severity of symptoms	Illiterate Literate	7.72 (3.84) 7.44 (3.56)	0.636	0.525
Coping strategies	Illiterate Literate	4.00 (1.37) 4.15 (1.40)	-0.955	0.340
Doctor communication	Illiterate Literate	12.57 (2.87) 14.03 (2.01)	-4.795	< 0.001
Mental health	Illiterate Literate	10.71 (4.29) 8.64 (4.84)	3.998	< 0.001
Quality of life	Illiterate Literate	5.74 (1.97) 5.72 (1.07)	0.168	0.866

Mean results for gender and study variables are presented in Table 3b. Significant results show that women have higher coping strategies (4.24 vs. 3.91, *p* < 0.001), less communication with doctor (12.86 vs. 13.36, *p* < 0.001), and suffer from greater problems related to mental health (9.98 vs. 9.14, *p* < 0.005) compared to men.

Mean results for literacy and study variables are presented in Table 3c. Significant results show that illiterate patients have less communication with doctor (12.57 vs. 14.03, *p* < 0.001), and suffer from greater problems related to mental health (10.71 vs. 8.64, *p* < 0.001) compared to literate ones.

### Correlation analysis

Pearson correlation results (Table 4) show that mental health is positively correlated with severity of symptoms (*r* = 0.288) and negatively correlated with coping strategies (*r*=-0.243), doctor communication (*r*=-0.258), and quality of life (*r*=-0.297).

**Table 4:** Correlation analysis between study variables

	Mental health	Quality of life
Mental health	1	-0.297^*^
Severity of symptoms	0.288^*^	-0.220^*^
Coping strategies	-0.243^*^	0.051
Doctor communication	-0.258^*^	0.016
Quality of life	-0.297^*^	1

* p<0.05

Quality of life is negatively correlated with severity of symptoms (*r*=-0.220). Though quality of life is positively correlated with coping strategies and doctor communication, the results are not significant.

### Structural equation model

The Chi square test (χ2 = 2.26, *p* < 0.05) and goodness of fit index (GFI = 0.965) demonstrated a good model fit. The alternate fit indices confirmed the acceptable fit of the sample (CFI = 0.983, AGFI = 0.950, RMSEA = 0.070).

**Fig. 3:**
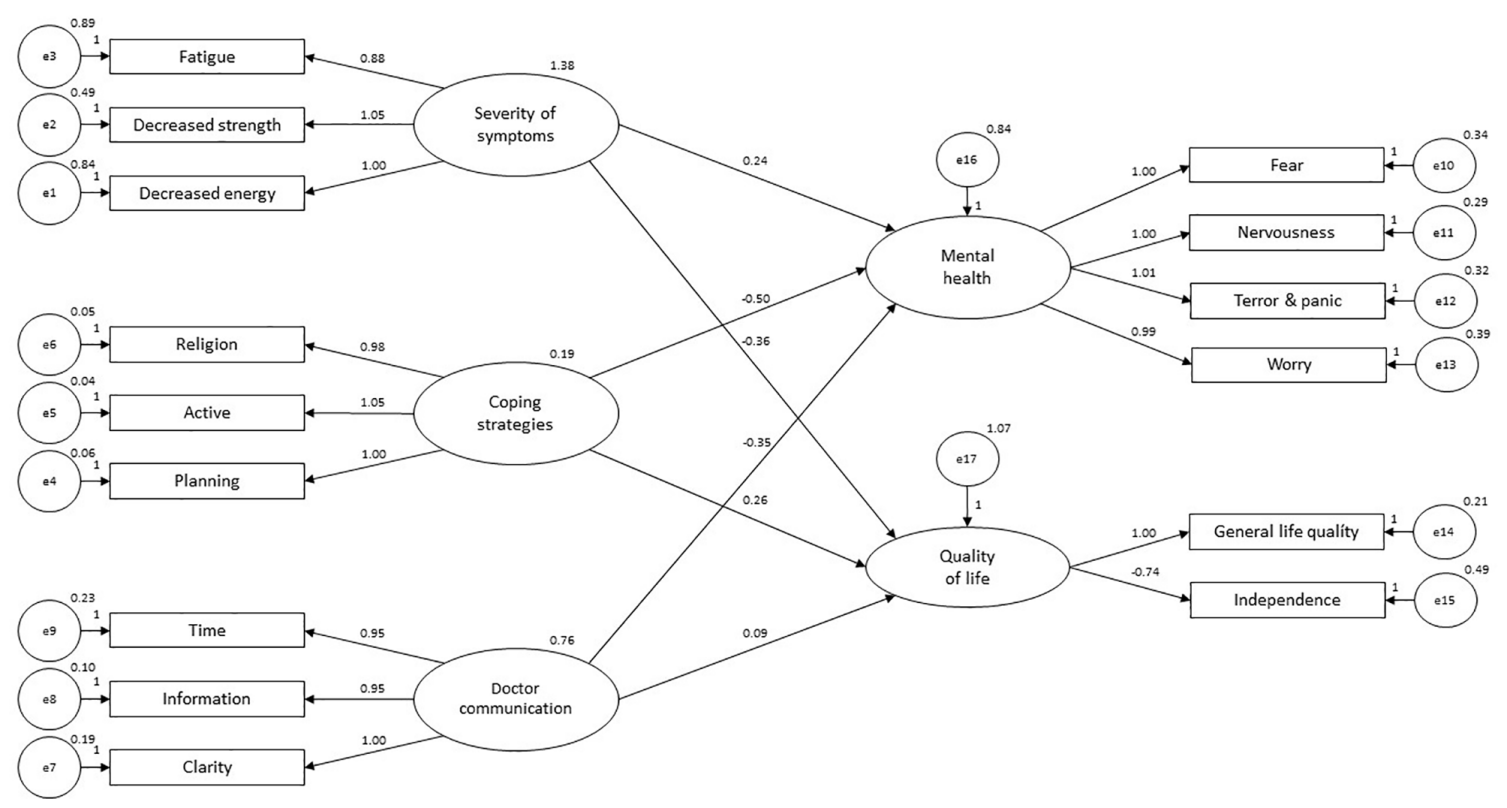
Structural equation model of severity of symptoms, coping strategies, doctor communication, mental health, and quality of life

All the coefficients between mental health and quality of life, and the three observed variables were found to be significant (Fig. 3). The results show that the three observed variables of severity of symptoms (β = 0.242, *p* < 0.001), coping strategies (β=-0.505, *p* < 0.001), and doctor communication (β=-0.352, *p* < 0.001), significantly explain mental health in patients of CLD. Similarly, the three observed variables of severity of symptoms (β=-0.363, *p* < 0.001), coping strategies (β = 0.258, *p* < 0.05), and doctor communication (β = 0.094, *p* < 0.05), significantly explain quality of life in patients of CLD.

## Discussion

With Pakistan’s high incidence of CLD burden, the public sector liver institute sampled in this study is playing a critical role in providing free and subsidized services to majority poor patients in the country who do not have out-of- pocket expenses [47]. Our findings show that women patients suffering from CLD have less communication with doctors and suffer from greater mental health challenges. It may be that women from more disadvantaged, semi-literate or illiterate backgrounds, and those belonging to conservative families have less ability to communicate and interact with physicians [48]. It may also be that doctors communicate less with female patients due to issues of ‘cultural conflict’, and perceptions that women complain more or misrepresent symptoms [49]. Other studies have also found that female CLD patients suffer from greater mental health disorders compared to male patients [50, 51]. This may be because women have less social support and face more stigma in Pakistani society when they suffer from a chronic disease [52]. Furthermore, women may also have more fears and anxiety when facing chronic disease burden due to their inability to continue care and nurturing for family, children, and other dependents [53].

Similarly, our results also show that illiterate patients suffering from CLD have less communication with physicians and suffer from greater mental health challenges. Other studies confirm that illiterate patients face problems in understanding medical vocabulary, reading prescriptions and physician’s instructions, and understanding patient education pamphlets and instructions for revisits and further tests [54]. A study conducted in Pakistan confirms that illiterate patients of CLD gain less information from doctor consultations and have low knowledge about how to manage disease and diet, consequently leading to adverse health results [55]. Another local study reports that illiterate patients suffering from CLD experience higher incidence of depression [56]. Anxiety and stress in illiterate populations of CLD has been found to be high due to lack of awareness and clarity, misconceptions and myths, and language barriers about disease [57].

Our results also show that men have fewer coping strategies pertaining to religion, active behaviour, and planning for disease management. Another study confirms that women are better at adopting coping strategies during illness, as they are more comfortable in showing emotions, turning to religion, and dealing with challenges by using social support [58]. Comparatively, men are less inclined to turn to religion or exhibit the emotions needed for adaptive coping during illness [59]. Patients of CLD are not always hospitalized or in doctor consultations, and thus development of self-care and coping strategies are integral to maintain their quality of life and mental health [60].

With regard to our first hypothesis, the study findings confirm that mental health of patients with CLD has a significant and positive relationship with severity of symptoms and a negative relationship with coping strategies and doctor communication. The second hypothesis of the study is also proven correct, and quality of life of patients with CLD has a significant and negative relationship with severity of symptoms and a positive relationship with coping strategies and doctor communication. Our findings confirm research from the developed world and that the management of patients with CLD is incomplete without interventions to improve and maintain mental health [22] and quality of life [61]. Based on the findings of this study, five areas need to be urgently addressed to improve the mental health and quality of life of CLD patients.

First, the management of severity of symptoms needs to be improved effectively through improved surveillance of high-risk groups at early stage and in primary settings [62]. Early treatment and effective lifestyle changes are needed for CLD patients of Pakistan [63], which is a partnership between the health sector, the patient, and their family. The support of the family and awareness and willingness of the patient to seek early treatment and commit to weight management, improved physical activity, and better nutrition can play an integral role in preventing advancement of symptoms [64]. Scholarship asserts that managing symptoms of CLD patients and preventing or delaying progression requires intensive surveillance and individually tailored therapeutic approaches, which is only possible through joint efforts by the provider, patient and support network including family and friends [65, 66]. In addition, a systematic literature review confirms the integral role of social workers or social policy officers in being integrated in the hospital team and managing the psychosocial aspects of CLD patients and progression of symptoms [67].

Second, CLD patients need to be supported for religious and spiritual coping, as well as in the development of active and planning coping behaviours. Numerous interventions which include emotional support, self-distraction, positive reframing, and acceptance [68] have been used to develop coping skills in patients with CLD, with the role of religious coping shown to be the most effective in Pakistan [69]. Developing coping strategies suited to the individual depends on early screening of personality behaviour and life circumstances and requires the support of a counsellor [70]. Recommended steps for developing coping behaviours in chronically ill people includes assisting patients in expressing grief, providing opportunities to seek personal meaning, and training to gain mastery in managing emotions and challenges, which are best achieved through a combination of support from the counsellor, social worker, provider, and family members [71]. Other research confirms that religious leaders and community notables can play an integral role in addressing challenges of the chronically ill and supporting patients with spiritual coping [72].

Third, training for doctor communication skills needs to be improved and maintained as a continued learning model. Studies show that doctors have less interest in continued learning for communication skills due to excessive workload and preference for expanding or updating medical knowledge [73]. It is thus important for health administration to ensure mandatory and continued training, and to ensure providers gain the relevant communication skills needed for different patients, as hepatocellular carcinoma patients may require different communication methods compared to patients with cirrhosis [74]. The unfavourable doctor to patient ratio, estimated at 1:1,300, also needs to be improved to support doctor communication and efficiency [75]. Supervision of doctor communication with patients by independent bodies in the hospital setting can be an important means of providing support to both the patient for better quality care and the doctor who can share needs and concerns about role burden or lack of administrative support [76]. Health sector regulatory bodies who have used patient satisfaction questionnaires to assess communication skills of providers, have been able to develop an effective action plan for improved healthcare services [77]. Social workers and social policy officers working in the hospital setting have been found to be useful partners in supporting doctors for improved communication and decision-making for patients [78].

Fourth, mental healthcare services and counselling of CLD patients must be introduced in the tertiary institutes of Pakistan [79]. Our study found that the prevalence of mental health disorders in CLD patients is very high, including feelings of worry, sadness, loneliness, and fear for the future. Other local scholarship confirms that mental health in CLD patients is a considerable challenge [80], but this study has determined statistical frequencies for different mental health disorders which highlight the need for early screening and management. Of great concern is that suicide ideation has been indicated by a significant number of CLD patients in our sample. Given that Pakistani society is known to underreport suicide due to religious and cultural reasons [81], it may be that suicide ideation prevalence is even greater than what is indicated in this study. Not only is integration of mental health services by counsellors essential at tertiary level for CLD patients [82], but there is need for mental health services to be designed according to the cultural and religious beliefs for improved uptake [83]. Social workers and social policy officers have been found to be effective in providing mental healthcare support to patients as they are trained to manage cultural differences and provide religiously sensitive services [84].

Finally, health literacy for CLD patients is integral as the disease is complex and even educated patients need awareness and support for disease management. As mentioned earlier, Pakistani CLD patients suffer from backgrounds of low literacy and there is no support for health literacy in the hospital setting. Studies have found that the integration of social case workers in the hospital team can improve health literacy in patients leading to better health outcomes [85]. Table 5 summarizes what has been discussed above and recommends a ‘bio-psycho-social-spiritual model’ to improve mental health and quality of life among CLD patients in Pakistan. This model can be managed by social workers or social protection officers in the tertiary setting, who are known to be successful patient case managers on interdisciplinary disease management teams [86].

**Table 5:** A ‘bio-psycho-social-spiritual model’ to improve mental health and quality of life among chronic liver disease patients in Pakistan

Intervention	Elements	Responsibility
Health literacy	• Literacy for CLD management • Specific and additional support for illiterate and semi-literate patients	Social officers
Prevent and manage severity of symptoms	• Early detection • Effective lifestyle changes	Provider / Patient / Support network and family
Coping strategies	• Active and planning coping • Religious and spiritual coping • Emotional coping	Counsellor / Support network and family / Religious notables
Doctor communication	• Continued training of providers • Linking professional advancement to patient surveys • Gender sensitization training	Health administration / Independent surveyors
Integrating mental health services	• Routine counselling for CLD patients	Counsellor / Health administration

## Conclusions

This study is limited to a single centre sample and is not representative of the entire nation. We recommend future studies to sample other provinces and to attempt longitudinal intervention-based research to assess the impact of improved care plans for patients with CLD. However, this study is an important contribution to the limited research about CLD patients who need support for mental health and quality of life, and it recommends the adoption of a holistic bio-psycho-social-spiritual model of care for CLD patients of Pakistan. Given that Pakistan is a developing nation with low allocation of health budget, inefficient governance and planning, and a large rural and impoverished population, it is imperative to design prudent interventions for CLD patients using culture-specific and self-care initiatives. Early treatment and effective lifestyle changes are essential to manage severity of symptoms. Mental health service models which are culturally appropriate and regionally accepted need to be considered. The study also highlights that female patients of CLD need support through female HCPs and female coordinators at health centres for better communication and compliance. Self-care programs and education for adopting coping strategies related to religion and spirituality, active behaviour, and planning for disease management and recovery are also needed. Education of medical students and continued training of physicians is needed for improved patient-doctor communication overall, and specific doctor training for gender sensitization and support for illiterate patients. Finally, the overburdened and understaffed healthcare provider teams at hospital settings and tertiary sector of Pakistan must be reinforced and made more efficient through the integration of social policy officers or social workers.

## Data Availability

Data is available from corresponding author upon reasonable request.

## Abbreviations

AGFI: Adjusted goodness of fit index
CLD: Chronic liver disease
CFI: Comparative fit index
COVID-19: Coronavirus Disease 19
GFI: Goodness of fit index
PKLI&RC: Pakistan Kidney and Liver Institute and Research Centre
RMSEA: Root mean square error of approximation
SD: Standard deviation
USD: US -Dollar
